# Calibrating canines—a universal detector calibrant for detection dogs

**DOI:** 10.3389/falgy.2024.1366596

**Published:** 2024-03-12

**Authors:** Michele N. Maughan, Jenna D. Gadberry, Caitlin E. Sharpes, Patricia E. Buckley, Aleksandr E. Miklos, Kenneth G. Furton, Lauryn E. DeGreeff, Nathaniel J. Hall, Robin R. Greubel, Katylynn B. Sloan

**Affiliations:** ^1^ Excet, Inc., Springfield, VA, United States; ^2^ Intrinsic24, LLC, Hayden, ID, United States; ^3^ Biochemistry Branch, U.S. Army DEVCOM Chemical Biological Center, Aberdeen Proving Ground, MD, United States; ^4^ Department of Chemistry and Biochemistry, Florida International University, Miami, FL, United States; ^5^ Formerly of the U.S. Naval Research Laboratory, Washington, DC, United States; ^6^ Department of Animal and Food Science, Texas Tech University, Lubbock, TX, United States; ^7^ K9Sensus Foundation, Lucas, IA, United States; ^8^ Technical Services Division, United States Secret Service, Washington, DC, United States

**Keywords:** scent detection, olfaction, universal detector calibrant (UDC), canine training, olfactory science

## Abstract

Since the advent of the Universal Detector Calibrant (UDC) by scientists at Florida International University in 2013, this tool has gone largely unrecognized and under-utilized by canine scent detection practitioners. The UDC is a chemical that enables reliability testing of biological and instrumental detectors. Training a biological detector, such as a scent detection canine, to respond to a safe, non-target, and uncommon compound has significant advantages. For example, if used prior to a search, the UDC provides the handler with the ability to confirm the detection dog is ready to work without placing target odor on site (i.e., a positive control), thereby increasing handler confidence in their canine and providing documentation of credibility that can withstand legal scrutiny. This review describes the UDC, summarizes its role in canine detection science, and addresses applications for UDC within scent detection canine development, training, and testing.

## 1 Introduction

Olfaction, the sense of smell, is a powerful and yet poorly understood sense. It is possible that it is under-studied due to the human bias towards visual and auditory senses. Practically, olfaction is more difficult to study when compared to visual or auditory systems, given that we have excellent tools (cameras, microphones, and powerful methods to quantify those data) with which to design and control visual and auditory studies. To do the same for olfaction would require the ability to perform time-resolved chemometric imaging at a scale relevant to a model organism, which is not possible at the time this manuscript was prepared. Even in situations where an olfactory study can be adequately controlled, the profound complexity of the olfactory system and its inextricable link with neurocognition complicates study design and interpretation. There is even complexity organizing the multi-disciplinary research teams needed to study olfaction as they often require physicists (to study odor movement), chemists (to understand odor profiles), biologists, behaviorists, and medical doctors (to understand the animal “sensor” at various levels), and highly specialized neuroscientists, geneticists, computer scientists, and mathematicians (to connect the dots). Much of this research did not begin before the seminal discovery by Buck and Axel (1991) of the multi-gene family of odorant receptors responsible for olfactory perception at the molecular level, which led to their 2004 Nobel Prize for Medicine (1).

Olfactory testing in humans has often employed a standard reference odor, 1-butanol, for the purposes of inter- and intra-subject comparisons (2–5). Similarly, researchers investigating odor and canine detection disciplines have utilized a variety of arbitrary/convenient compounds to investigate detection limits, odor capacity, and odor memory. These studies have often used compounds like n-amyl acetate (6, 7), spices/extracts (8–11), and perfluorotributylamine (PFTBA) (12). While advantageous for their intended research purpose, ultimately these compounds are too commonly encountered in the natural environment or too expensive for everyday use. For example, n-amyl acetate is found in flavorings, nail enamels, perfumes, and photographic films. Since detection canines used in criminal investigations are often used for probable cause, responding to a non-target compound in an operational search is unacceptable. While there is research indicating that detection canines can discriminate between target odor and innocuous items that may contain a component of the target odor (13), most organizations are risk averse to purposely training canine teams on commonly encountered odors.

Typically, a training aid substance is selected early in a detection canine's development, which will then determine that dog's future career. For example, when training a drug detection canine (DDC), trainers often “imprint” the canine on the odor of cocaine as a first exposure to odor detection. This step essentially commits that canine to becoming a DDC, due to the fact that it may involve significant time to confidently extinguish if the dog changes discipline trajectories. Studies have shown that even in the absence of exposure to the originally trained odor, a canine's odor memory spans over twelve months (9, 10, 14). To that end, it could be subject to legal challenge if a dog trained on odors from one discipline was later transitioned to another, such as going from human remains detection to drugs/narcotics. While it is possible, this example illustrates why this approach is fraught with safety and legal concerns and is not considered to be best practice. In another example, an explosive detection canine (EDC) handler must be certain their canine responds to the odor of explosives and does not also respond to drugs because the law enforcement and security response to illicit drugs is very different than to explosives. In this example, an evacuation of the area and response of an explosive ordnance disposal (EOD) team would be unnecessary if the dog unknowingly alerted to a previously trained drug odor.

What if a trainer did not have to commit their young canines to a specific detection discipline? In this case, having an odor that is uncommon in operational environments, easy to control, safe to handle and behaves similar to most trained targets would be a valuable tool to train, validate and calibrate detector canine performance. Such a UDC would be a useful tool in teaching young canines to learn to detect an odor, search for this odor in the environment, and demonstrate a trained final response (TFR) to communicate to the handler that the target odor has been located. A TFR is defined by the American Standards Board as, “A behavior that a canine has been trained to exhibit in the presence of a target odor/scent source. This behavior may be either passive (sit, stare, down, point, etc.) or active (bite, bark, scratch, jump, etc.)” (15). 1-bromooctane (1-BO) specifically is ideal as a UDC because it is not known to be present in any drug, explosive, or other canine training aid odor profile. It is typically only utilized in organic synthesis and solvent extraction reactions and hence is quite rare in the natural environment.

The UDC is a chemical that enables reliability testing of biological and instrumental detectors. The value and benefits of a UDC are not limited to canines in law enforcement roles. With the expansion of detection canine disciplines addressing everything from contraband (e.g., currency) to forensics (e.g., accelerants), search-and-rescue (e.g., live or deceased humans), and the increasing use of canines in biomedical detection (e.g., COVID), canines are engaging in detection of a burgeoning list of substances (16). These canines serve vital roles in situations where there is no current method of detection or where the area to be searched is simply too vast to be practicably searched by humans. The UDC can be used as a detection teaching tool that does not commit a canine to any specific discipline.

In this review, we discuss and use the term “UDC” in two ways: as “the” UDC down-selected to single chemical odorant, 1-BO, and as “a” UDC in which we leave the door open to the inclusion of other UDC candidate odors should they be identified or created in the future.

## 2 Universal detector calibrant

**Figure 1:**
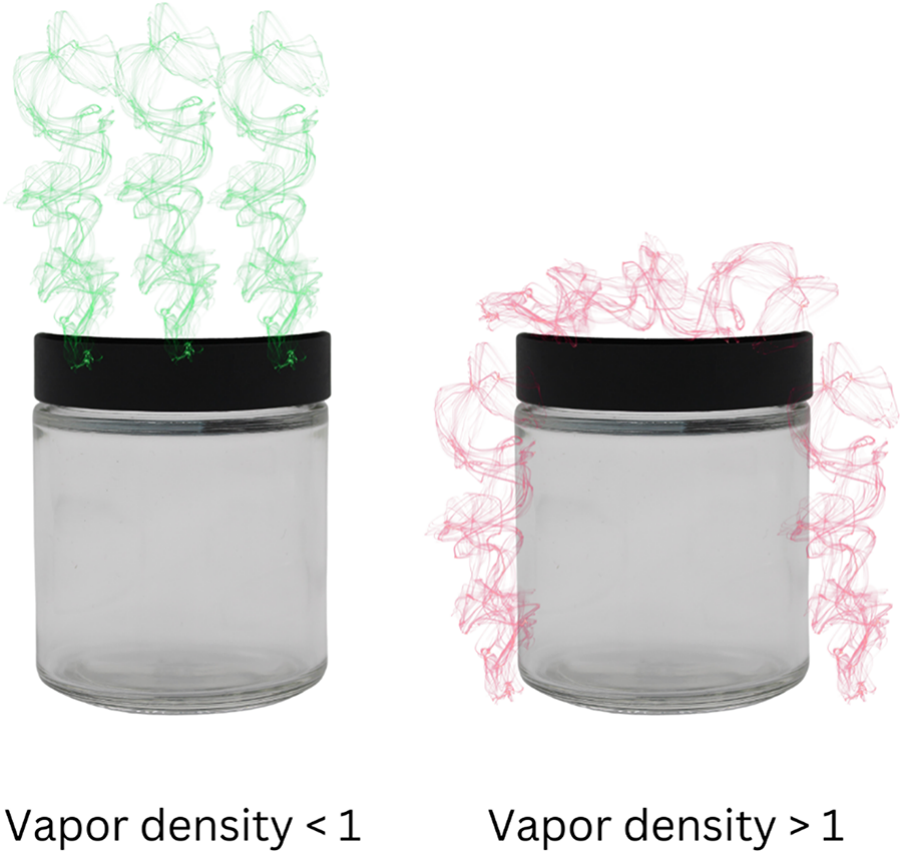
Vapor density illustration. On the left is a depiction of a substance with a vapor density less than 1 (air = 1) and therefore the gasses will rise. On the right is a depiction of a substance with a vapor density >1 and therefore the gasses will sink. 1-BO has a vapor density of 6.67 (i.e., >1) and thus will off-gas similarly to the image on the right.

The Universal Detector Calibrant (UDC) is a single chemical odorant used to calibrate a biological detector. We propose it should be volatile, detectable by the olfactory system, scarce in environment, non-toxic and stable. Currently the only widely tested and commercially available UDC is 1-Bromooctane (1-BO) (CAS # 111-83-1). Other UDCs including halogenated alkyl, aryl and thiol compounds, epoxides, ketones, esters, and aldehydes with specific properties stated above are being researched to increase the suite of potential UDCs. 1-BO is a colorless to yellow liquid with a vapor pressure of 0.34 mmHg at 25°C (77 °F). It has a molecular weight of 193.2 g/mol; compared to air (molecular weight of 28.97 g/mol) it has a vapor density of 6.67, meaning 1-BO vapor is 6.67 times denser than air, therefore 1-BO is less buoyant than air and will sink (See Figure 1 for an illustration).

It is important to note that 1-BO odor behaves similarly to most canine training aid odors in that it is negatively buoyant (i.e., heavier than air); This should hold true for any UDC candidate. However, the factors that determine odor movement more than any inherent physical or chemical property of the substance are advection (a macroscopic process that moves and disperses gas due to the ambient motion of air) and diffusion, with the former having a greater effect on vapor distribution than diffusion in most real-world scenarios. Air currents, such as convective turbulent air flows, move odor molecules around in space rather quickly, whereas diffusion is responsible for the mixing of gases as they follow concentration gradients. Taken together, 1-BO will behave similarly to other canine training aid odors, which is an important feature of a UDC. This feature allows canine trainers to utilize a UDC in training comparably to how they use their other training aid odors and similarly it allows the canine to follow odor plumes typical of what they will encounter in the real world. If a UDC odor behaved very differently than other canine training aid odors, this could affect the way canines search and could negatively impact the efficiency and effectiveness of the search.

## 3 Criteria for and characteristics of a UDC

The criteria for and characteristics of a UDC are summarized in Table 1 (17). The criteria are divided into mandatory and desirable categories. The mandatory criterion for a UDC is first and foremost, that it poses minimal health hazard to the canine and handler. Detection canines are often subject to an array of occupational hazards because of the threats they encounter in operations and because of the intensity of the training they undergo in preparation. Detection canines also face several inhalational and ingestion hazards based on the fact that many of their training aids are hazardous to respire or ingest. When instituting another odor for the canines to be trained on, it is important that we are not adding to the cumulative lifetime occupational risks to the canine.

**Table 1:** UDC selection criteria, description, and considerations for odor delivery. Adapted from Beltz (2013).

UDC Criteria	Criteria Description
Mandatory	
Minimal health hazard	The chemical should present minimal health hazards since a UDC training aid is designed to be used daily by canines and handlers. Chemicals selected should be only classified as irritants with no special transportation, disposal, or special handling requirements determined by the chemical's National Library of Medicine's PubChem data.
Non-discipline specific odor	The chemical may be used for explosives, narcotics, human remains, search and rescue, electronics, invasive plants or animals, conservation of endangered plants and animals, human tracking, cancer, or infectious diseases detection canines. Because of this wide variety of potential discipline-specific odors, the chemical should not be part of a common odor that any of these detection canines may encounter over their career.
Stable	The chemical will be used in a wide variety of indoor and outdoor environmental conditions during storage and training; therefore, the chemical should be resistant to degradation under varying conditions that would potentially change the volatile organic compound's profile.
Commercially available	The chemical should be available from multiple suppliers. Another alternative is that the training aid could be conveniently prepared to make it commercially available such as taking the primary chemical and adding a carrier compound such as cellulose.
Rare in the operational environment	The chemical should not be commonly found in the environment or used in a wide variety of products. This limits the possibility of a detection canine false response while training or on missions in diverse indoor and outdoor environmental conditions.
Volatile	The chemical should readily enter and be detectable in the vapor phase by canine or instrument. The chemical should have one to 20 carbon atoms, or a recommended vapor pressure of at least 10^−7 ^mmHg and a boiling point of less than 325 °C.
Detectable by detection canines	The chemical should be detectable by detection canines, i.e., the canine's olfactory system will have a sufficient olfactory threshold for detection of a UDC under operationally relevant vapor concentrations.
Low chemical reactivity	The chemical should not react with any commonly used materials. This allows for fewer limitations on testing, storage, manufacturing, training, and odor delivery device configurations. For example, corrosive chemicals are not suitable as they present health and safety issues.
Desirable	
Low cost	The chemical should be affordable for the canine detection community. Funding is of concern for any canine detection program. The final cost will be determined by the chemical chosen, the amount required, and the odor delivery mechanism used.
Detectable by other animals including humans	This allows the handler or other personnel to perform a quick “sniff” test themselves and easily determine if a UDC training aid lacks odor or if the odor has changed, thus signaling the need for a new UDC training aid.
Readily detected by instruments	The chemical should be identifiable by mass spectrometry, as well as a wide variety of other laboratory and portable instruments and be soluble in commonly used solvents.
Consistent odor release	The chemical should give off odor at a constant rate by itself or through the configuration of the odor delivery device used to make a UDC training aid.
Limited to no environmental hazard	The chemical should not present a hazard to the environment and if limited evidence of hazards exists, the chemical should be disposed of in accordance with local, state, and federal regulations.
Compatible with multiple form factors and delivery mechanisms	The chemical should be compatible with a variety of form factors and delivery mechanisms according to the intended use. This allows the end user to package the chemical in multiple configurations.

A UDC must also be a non-discipline specific odor, i.e., an odor outside the suite of their future discipline specific odors (e.g., narcotic, or explosive). Often times this odor is referred to as “non-target”, however, in the case of a UDC, this odor becomes a target odor through training, therefore “non-discipline specific” is the more accurate term. This criterion allows for the UDC to be used by any canine and by any canine destined to become a detection canine. In addition to non-discipline specific, a UDC must also not contain any odorants that could be present in future target odor profiles so that detection canines do not respond to an explosive or other chemical substance with a UDC in its odor profile.

A UDC must be stable in the environment. Detection canines train and operate in a wide variety of environmental conditions with varying temperature, humidity, precipitation, pressure, etc. and therefore a UDC odor profile must be relatively unaffected by these changing conditions. If a UDC odor profile were to degrade under variable weather conditions, such as increased temperature or exposure to air or humidity, could potentially alter the odor profile of a UDC, impacting the effectiveness of the aid.

A UDC must be readily available and ideally readily commercially available. This is a critical issue in the detection canine industry: the availability of training aids. Ideally, there would be multiple manufacturers to avoid supply chain issues, or situations in which handlers do not have consistent access to a UDC training aid. 1-BO is produced by several chemical companies, however, its sale is restricted to registered laboratories with validated shipping addresses. This constraint limits the UDC's availability as a canine training aid to the general public, however, it does ensure that proper precautions are taken with respect to storage and handling of the bulk liquid 1-BO. As it stands, 1-BO is commercially available: Redland Ahead UDC Canine Training Aid Package (Innovative Detection Concepts, Miami, FL).

A UDC must be rare (or non-existent) in the operational environment alleviating the risk of the canine responding to it in the operational environment and causing nuisance alarms. A nuisance alarm occurs when a detection canine reports a valid detection of a trained odor, that is not associated with the targets of interest, having no security or operational implication. An example of this is an EDC response to medically prescribed nitroglycerin pills carried by a person. In this instance, because nitroglycerin is present in many forms of dynamite, when a canine responds to the medication it is not a false response because the target odor is actually present; but there is no relevant threat. This being said, the canine response is detrimental to operations as the handler will not necessarily know that this is a nuisance alarm (and not the canine responding to a real threat material) and will enact a series of actions (e.g., police search and seizure or calling the bomb squad) that can undermine trust in future responses from that canine. Ensuring that a UDC is unlikely to be encountered during training or operations increases handler trust and detection reliability as we can be confident that a UDC odor is not causing the canine response. To that end, training with a UDC fortifies the probable cause mandate for legal search and seizure.

There is a clear tension between readily available and rare in the operational environment, in that as use of the UDC increases, so would the odor of UDC in the operational environment. It is therefore important to continue to use good training aid storage and handling protocols to avoid contaminating the training/operational environments with target odor. This also speaks to the need for additional UDC candidate odorants so that if unintentional contamination occurs, an alternate UDC could be utilized instead.

A UDC must be sufficiently volatile. Volatility, in this case, refers to the vapor pressure of a chemical and the likelihood for it to be in the gaseous phase. If a UDC is not sufficiently volatile (i.e., its vapor pressure is extremely low), then odor availability is limited and not enough of a UDC odorant will enter the canine's nasal cavity, making detection difficult, which would hinder the detection learning process. On the other hand, if a UDC is too volatile (e.g., acetone), then it will evaporate too quickly into the environment such that it would not make a viable training aid as it would have a short shelf- and service-life limiting its utility as a training aid.

A UDC must be detectable by detection canines. This seems like an obvious criterion; however, it is important to note that—just like the electromagnetic spectrum of wavelengths, of which visible light is a small portion that humans can visually sense—there are odorants that humans (or other animals) cannot smell. Any UDC candidate odorant must be detectable by canines and be a conspicuous odor. It should also be taken into consideration that animals have differing olfactory thresholds for different odorants, and there is great variability amongst canine breeds, sex, age, and detection training protocols (18).

Finally, the last mandatory UDC criterion is that it must have low chemical reactivity. The more reactive a chemical is, the fewer options for its storage, transportation, and handling. Highly reactive chemicals also tend to present health and safety hazards that would preclude their use as a UDC. Low chemical reactivity provides assurance that the UDC candidate odor can be reasonably contained and utilized without creating additional risks to the health and safety of the canine and handler, and also enables the shipment of UDC training aids via air, land, and sea, thereby lowering the logistical burden of trying to get a UDC from the manufacturer to the end-user.

There are five desirable characteristics of a UDC. Some may be considered mandatory or may be irrelevant depending on the needs of the end-user. The desirable criteria are that a UDC be 1) low cost (affordable), 2) detectable by other animals including humans so that the handlers can smell a UDC at its higher concentrations to verify the odor's presence, 3) readily detectable by instruments so that the presence of UDC odor can be verified and potentially quantified by another detection instrument/technology, and 4) provides consistent odor release so that canines can encounter similar amounts of odor regardless of when they train on a UDC or how old it is, and 5) compatible with multiple form factors and delivery mechanisms to allow the end user to utilize their preferred packaging and delivery device. This last criterion deserves additional explanation and will be discussed in Section 3.

## 4 UDC odor delivery mechanisms

As it is utilized presently, the UDC is or can be packaged in the controlled odor mimic permeation systems (COMPS) consisting of target odorants sealed in a permeable polymer bag (19, 20), the training aid delivery device (TADD®) (SciK9® LLC, Lorton, VA) (21, 22), absorbed onto cellulose filter paper (23) (Redland Ahead UDC Canine Training Aid Package, Innovative Detection Concepts, Miami, FL), or adsorbed onto a Getxent (Neuchâtel, Switzerland) tube (24). The concentration of UDC vapor can be systematically varied to assist canines in recognizing the varying amounts of odor they will encounter in search scenarios and assist them in generalizing from “trace” to “bulk” amounts of odor. It should be noted that 1-BO is a flammable liquid and “very toxic to aquatic life with long lasting effects” per the Safety Data Sheet (25). Typically, in laboratory environments, 1-BO is stored in a special flammables cabinet, disposed of in the biohazard waste stream, and handled with proper personal protective equipment (PPE) and in a fume hood. Once the UDC is diluted and in its odor delivery device, it no longer requires the precautions associated with the bulk liquid material.

**Figure 2:**
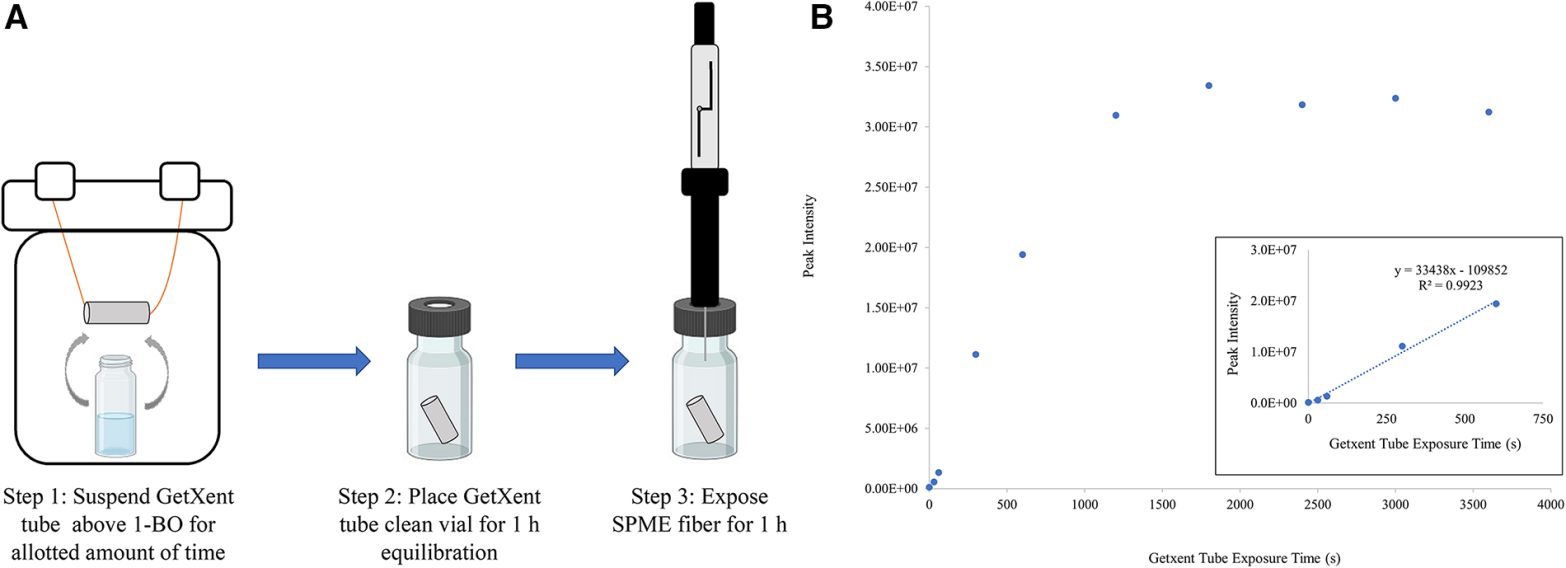
Preliminary study of UDC emission from getxent. (**A**) Representation of Getxent tube being exposed to 1-BO in the vapor phase, followed by equilibration and headspace extraction by a divinylbenzene/polydimethylsiloxane/carboxen solid phase microextraction (SPME, Millipore Sigma) (**B**) Data points represent the abundance of 1-BO collected from the headspace of Getxent tubes after having been exposed to neat 1-BO for increasing time (s). The inset reveals the linear portion of the same graph. Following extraction, the SPME fiber was then extracted and thermally desorbed (250 °C) in the inlet of the gas chromatograph (GC) equipped with a 30 m × 0.25 mm ID × 1.0 mm df Restek RTX-Volatiles column, followed by detection by mass spectrometry (Agilent 7890 GC / 5,977 MSD). The flow rate was set to 1 ml/min with a 10:1 split ratio. The GC oven was initially heated to 70 °C for 1 min, followed by an oven ramp of 25 °C/min to 240 °C where it was held for 1 min until completion.

The benefit of the UDC (5 μl) absorbed onto a cellulose filter paper as provided by Redland Ahead (Miami, FL) is that this form factor provides a single-use UDC training aid, which greatly simplifies detection training from an inventory and tracking perspective and limits environmental contamination associated with repeated use. The drawback of this form factor is that the training aid liquid can contaminate the training environment with 1-BO odor and there is no control over odor emission rate as the 1-BO will quickly evaporate off the paper. Getxent tubes adsorbed with UDC odorant can be single-use or re-used for a limited amount of time after the initial use (assuming proper storage conditions). The Getxent tube has the added benefit of providing an adjustable level of odor, i.e., the longer the tube is co-incubated with odorants to “load” the tube, the emission rate of those odorants increases. This, however, is caveated by the fact that each odorant has a different adsorption and emission rate and the tube will inevitably reach a saturation point (26). In a small preliminary study, a Getxent tube was suspended above a vial containing several milliliters of neat 1-BO together in a 500 ml perfluoroalkoxy (PFA) jar (Figure 2A, Step 1). Getxent tubes were exposed to the UDC for varying amounts of time, 30 s to 1 h. Following exposure, the tube was placed in a separate vial, allowed to equilibrate (Figure 2A, Step 2), and the resulting 1-BO in the headspace was sampled by solid phase microextraction (SPME) (Figure 2A, Step 3) with analysis by gas chromatography / mass spectrometry (GC/MS). These preliminary results showed that the abundance of 1-BO collected from the headspace of the tubes increased linearly with increasing exposure time from 30 s to 10 min. Additionally, exposure time beyond 10 min produced no significant increase in abundance (Figure 2B). These data indicated the potential for using the Getxent tubes with the UDC to lower detection threshold by reducing the abundance of odor systematically.

Out of the aforementioned odor delivery mechanisms, two are able to precisely vary the concentration of UDC presented to the canine by altering the emission rate of 1-BO. This capability is important because recent data suggests that canines must be trained on varying concentrations of a target odor in order to find large/bulk quantities and small/trace amounts of odor operationally (27–30). The COMPS form factor allows one to package the UDC liquid absorbed onto a substrate into permeable polymer bags of different thicknesses with permeation rates declining as the thickness of the bag increases. The Training Aid Delivery Device (TADD®) jar with its microporous membrane, allows for the diffusion of UDC across the membrane and one can change the concentration by diluting it with cellulose powder or by decreasing the surface area of the membrane using odor restriction caps. When the UDC-cellulose mixture is in the TADD®, the training aid is protected from environmental contaminants and the training area is protected from residual odor.

**Figure 3:**
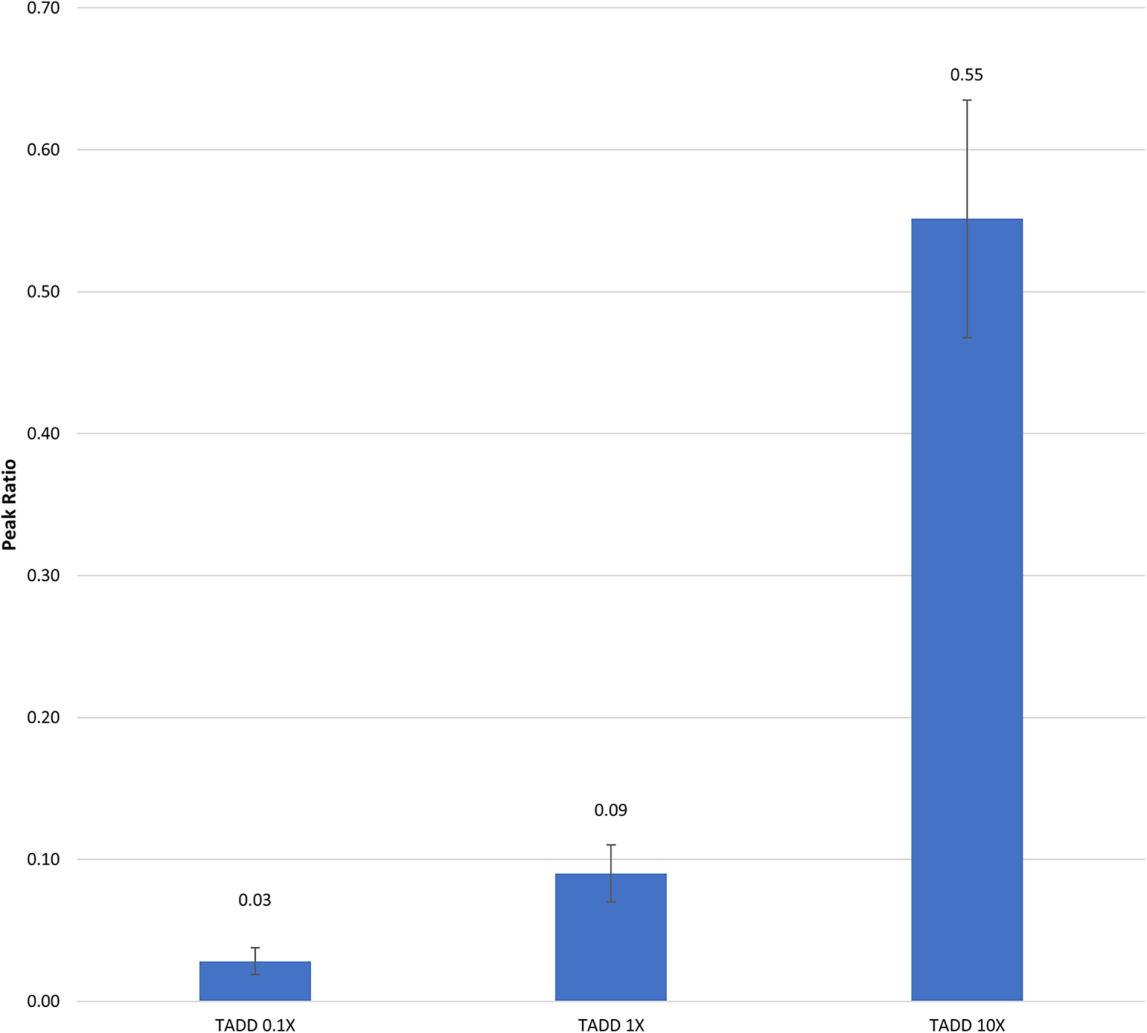
Semi-Quantitation of differing concentrations of 1-BO in a TADD®. The bars represent the relative peak areas of 1-BO vapor emanating from TADDs®. The TADDs® were placed in 500 ml PFA jars (Savillex) for 1 h, after which 1-BO vapor was collected from the headspace by exposing a divinylbenzene/polydimethylsiloxane/carboxen solid phase microextraction (SPME) fiber (Millipore Sigma) to the headspace of the jar for 1 h. The fiber was then extracted and thermally desorbed (260 °C) in the inlet of the gas chromatograph (GC) equipped with a 15 m, 0.32 mm ID 5 mm df Restek RTX-Volatile Amine column, followed by detection by mass spectrometry (Agilent 6,890 GC / 5,975 MSD). The flow rate was set to 3 ml/min with a 10:1 split ratio. The GC oven was initially heated to 40 °C for 1 min, followed by an oven ramp of 30 °C/min to 240 °C where it was held for 2 min until completion.

In DeGreeff et al. 2024 (31) the UDC in TADDs® was used in canine testing. Prior to testing, headspace analysis and semi-quantitation of the UDCs to be used were carried out. The UDC training aid used in the study consisted of 1-BO impregnated onto cellulose powder at varying concentrations: 100X, 10X, 1X, 0.1X, 0.01X, 0.001X, and held in a TADD®. The relative quantities of 1-BO from the headspace of the TADDs® was measured by Solid Phase Microextraction (SPME) GC/MS. The relative quantity of 1-BO was reported as the peak area of 1-BO in ratio to the peak area of an internal standard (1,5-dibromopentane) (Figure 3). Once the relative odorant quantities of the UDC in training aid delivery devices (TADDs®) were validated, they were then employed in several of research, development, test, and evaluation (RDT&E) projects carried out by U.S. Army Combat Capabilities Development Command (DEVCOM) Chemical Biological Center (CBC), Aberdeen Proving Ground (APG), Maryland. 1-BO is not water soluble, and is soluble in highly odiferous solvents (e.g., alcohol and ether) and non-odiferous solvents (e.g., mineral oil and diethyl phthalate). The former should be avoided to avoid adding extraneous odors to a UDC training aid and many odor delivery mechanisms are not compatible with liquid training aids, therefore diluting the UDC with a powdered substrate such as cellulose enables the creation of lower concentrations of the UDC than what could be physically pipetted without adding a liquid diluent. The use of cellulose powder as an odor impregnation substrate is quite common amongst the commercial off-the-shelf canine training aids. This is due to 1) the minimal amount of odor cellulose contributes to the overall training aid and 2) the powdered form factor creates a large surface area leading to greater odor emission and often renders explosive training aids non-detonable.

With any odor delivery mechanism, it should be noted that permeation or diffusion rates will differ depending on the odorant, as well as the type of substrate and barrier used due to inherent chemical properties of each odorant, such as vapor pressure, vapor density, and adsorption coefficients between the chemical, (e.g., 1-BO) and the barrier (e.g., LDPE). Certainly, more research is needed to determine the dissipation rates, shelf-, and service-life of 1-BO in a variety of odor delivery devices so that the end-user has guidelines to follow regarding usage, training time, and when disposal is warranted.

## 5 Applications of the UDC in training

There are many applications of a UDC as a training tool for canines of all ages and experience levels. Here, we describe the role of “a” and “the” UDC in various aspects of canine training. Some applications discussed herein are based on educated inference based on review of published knowledge and some applications are based on experience. All other applications have been explicitly tested by researchers and are referenced accordingly.

### 5.1 Early olfactory enrichment

Odorants can used be used for the early olfactory enrichment of young canines (32). Early olfactory stimulation research has been conducted for many years in rodents and points to the importance of olfaction in early brain development (33–36). Studies using rats demonstrate that early odor enrichment enhances their ability to discriminate components in binary mixtures that had not been discriminated prior to the enrichment period, and that this increase in discrimination capability extends beyond the specific odorants used during the enrichment period (33).

It is well-recognized that experiences during critical periods of early development significantly impact cognitive development in canines (e.g., 37). While many early intervention strategies have been tested in the canine population with the goal that we may better be able to predict and select working dog candidates, the effect of an olfactory-based early intervention is not well-studied. Why should there be a specific strategy for olfaction? Perhaps it is because there is a clear linkage between olfaction and cognition. The link is strengthened by the National Science Foundation's (NSF) Odor2Action initiative which has dedicated tens of millions of dollars to answer a fundamental question in neuroscience: How do animals use information from odor stimuli in their environment to guide natural behaviors? (38).

This question is being explored using a multidisciplinary approach in sensori-motor circuits, active sensing, and sensory coding, and should reveal new linkages amongst olfaction, the brain, and behavior. There are extensive connections of the canine olfactory pathways that have been elucidated by magnetic resonance imaging (MRI) and dissection techniques. These physical pathways provide insight into how canines integrate olfactory stimuli into their cognitive functioning (39). Andrews et al. recently found five olfactory tracts structurally linking the olfactory bulb directly to the following parts of the brain: occipital, piriform, limbic, cortical spinal, and entorhinal (39). These tracts provide an information highway to the parts of the brain responsible for vision, memory, emotion, motor function, olfactory processing, and executive function. Taken together, we get an appreciation for how extensively connected olfaction and cognition are within the canine brain. Stimulating olfaction early in canine life is therefore considered a critical step in not only the development of olfaction, but also brain development (40). This early enrichment can be accomplished in a variety of ways, however the two best described are: early enrichment and early imprinting. Olfactory enrichment involves the purposeful addition of specific odors to the environment of an animal to stimulate positive behaviors, while olfactory pairing involves providing positive reinforcement training whereby the canines are rewarded for performing scent discrimination tests (32). While the sample size was too small to make any conclusions about early odor exposure or training, it was found that canines in the early pairing group may demonstrate higher motivation for reinforcement relative to the control group, however, the sample size would need to increase to assess the validity of these initial findings (32).

### 5.2 Nonspecific detection training

The UDC can be used to teach canines proper search mechanics and a TFR before the canine is committed to a scent-specific discipline, such as narcotics or explosive detection. We reviewed in the Introduction, the many reasons that we preclude explosives and narcotics detection canines from switching disciplines, so the importance of not committing a canine to a scent-specific discipline during early training cannot be understated. Indeed, the weight of the decision to commit a canine to either/or disciplines is substantial. Therefore, the ability to train a young canine to search for, locate, and respond to a UDC odor without pre-determining the canine's future scent discipline, is tremendously valuable. Behaviors can be shaped, independent and systematic searching can be reinforced, and TFRs can be refined all by using the UDC instead of odors that 1) may be hazardous to the canine's health and 2) may vary in odor profile depending on the manufacturer or age of the substance.

Canines also perceive odors as representing specific objects, whereby if they smell a Kong® and are presented with a tennis ball in a violation-of-expectation experiment, they show “surprise” (41). Since canines are capable of metacognition and are able to “learn how to learn”, these abilities support the use of a UDC to teach canines how to learn in general and how to learn to search, locate, and report specifically. This use of the UDC is supported by the finding that training time is decreased for teaching canines subsequent odors after they have learned their first odor (11).

### 5.3 Decrease in discipline-specific training time

Prior research has indicated that once canines are “imprinted” or trained (or “paired”) on their first odor, the time required to train subsequent odor discriminations is shortened (11). Further, the UDC would allow for the introduction and training of various non-target/distractor odors that any operational dog may encounter in their operational environment. This would allow for substantive training of the various non-target odors they may encounter, potentially reducing potential false positives in the future. Further, we hypothesize that this experience with the mechanics of odor discrimination learning (e.g., where to search, how to search) using the UDC discriminated from various non-target odors could lead to a learning-set formation, whereby novel discriminations can be learned substantially faster (e.g., 39–41). This would allow dogs to receive nearly all training for detection work prior to committing the dog to a specific discipline, with minimal time to transfer this behavior to the discipline target.

### 5.4 No extinction training required

**Figure 4:**
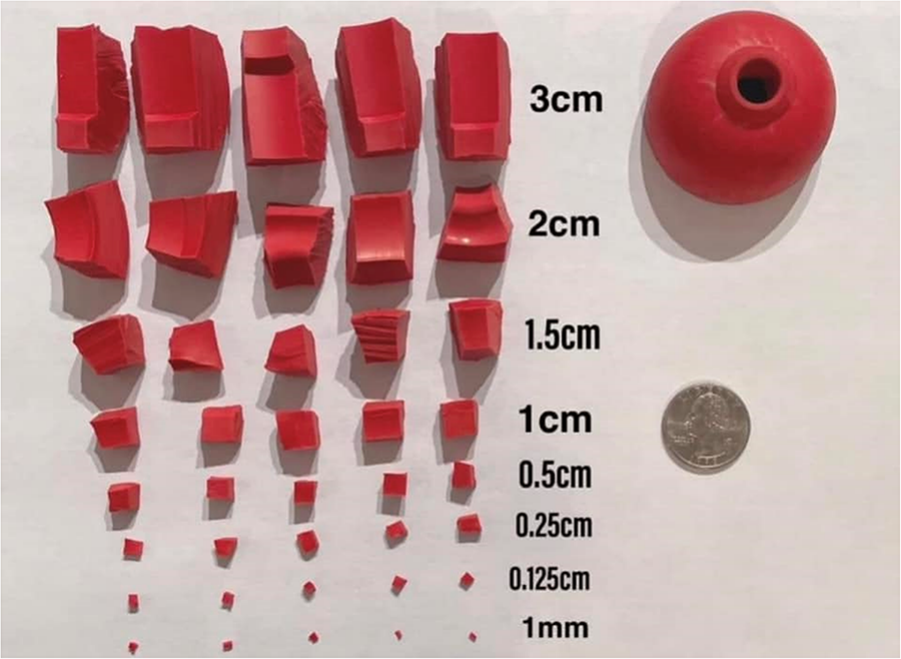
Kong® rubber canine toy. This toy is used as a reinforcement tool and odor source for training canines how to search-locate-report. Once the canine understands the detection process, the toy is often cut into increasingly smaller pieces to challenge canines’ thresholds. Image courtesy of Emma Schuett, CPDT-KA and re-printed with permission from Brush Education, Inc.

A method commonly used by detection canine trainers is the use of a rubber Kong® canine toy as not only a canine's reinforcer, but also the first odor used in scent detection training. Once the canines are proficient in finding the Kong®, it is often cut into smaller pieces to challenge the canine's detection sensitivity (Figure 4). However, once canines enter the operational environment, the Kong® method of odor training frequently proves problematic as the Kong® is made out of materials that are commonly found in the environment and may cause nuisance alarms by the canines. Additionally, trainers often have to “proof” canines off (or extinguish) responses to the Kong® odor due to their widespread use. By utilizing the UDC, instead of the Kong® for this initial odor training, canines will not have to undergo later extinction training.

### 5.5 Amenable to stimulus fading

The controllability of the UDC concentration to very high and below threshold concentrations allows for the flexibility to use various stimulus fading training procedures. During initial acquisition, odor detection training can be arranged in a format whereby a higher UDC concentration is used as the S+ (target odor) and a non-target stimulus (or stimuli; S-) is presented in a faded way such that few or no responses are made during the acquisition of this discrimination (i.e., “errorless” learning). Prior research has demonstrated various benefits for acquisition of a discrimination in this errorless method (e.g., 42–44) such as minimizing “frustration” related behaviors from the learner. An errorless approach, however, may not be readily possible with some discipline specific targets, making the UDC an ideal candidate for initial training. For example, some discipline specific targets such as oxidizer salts or TNT may have little odor availability with minimal initial salience for the dog. Thus, target odor stimulus intensity for these targets may not be able to be manipulated in such a way that only responses occur to the S+ (target odor) with no S- responses.

Additionally, after initial acquisition training, stimulus fading can be used to facilitate transfer to more challenging discipline specific target odors. In the early pigeon discrimination learning work, Terrace showed that novel visual discriminations can be acquired in an errorless manner through transfer from a previously learned discrimination (45). Terrace superimposed previously learned stimuli on the novel discrimination and slowly faded the original stimuli (45). An example application of this procedure with a UDC, would be following initial training with the UDC, the UDC and a discipline specific target could be presented together (superimposed). Through the easy manipulation of UDC concentration, the UDC would then be slowly faded, leaving only the discipline specific odor. If similar to the prior work with pigeons, such a procedure could allow for transfer to the challenging discipline specific odor with minimal errors and “frustration” or emotional responses from the learner that would otherwise occur following incorrect responses in a “trial and error” approach. A fruitful area of future research would be to systematically evaluate the effects of such training procedures and what impacts it might have on learner behaviors and overall responses to non-target (S-) odors (i.e., false alerts).

### 5.6 Vigilance and avoiding search extinction

The UDC can be used to provide canines with the opportunity to detect a target in their operational environment. For many detection canine disciplines, the prevalence of targets (e.g., the frequency of actual explosives being found for an explosive canine) is very rare. Canines can quickly learn the context and conditions in which the frequency of targets differs from training scenarios (46–48), and search behavior extinguishes (i.e., searching becomes non-productive and decreases)*.* In many cases, there are restrictions to placing live target materials (e.g., explosives) in operational environments for various safety concerns, which exacerbates this decline in canine search behavior and performance (i.e., 47, 48). Although training methods can help build the duration of search behavior in the absence of targets (i.e., 49, 50), being able to provide targets that can be placed in operational environments can be very helpful to maintain search performance.

The UDC may serve to help maintain search behavior and detection performance. Because UDC is non-hazardous and rare in the environment, there are minimal safety concerns to plant or “drop” UDC target odors in an operational environment for canines to find, assuming proper retrieval, storage, or disposal at the end of the training session. Recent research has demonstrated that training canines to find an irrelevant odor, that can be “dropped” or placed in operational environments can have important impacts in maintaining canine search behavior in operational environments (47) and in a rodent model of detection canine performance (14). Furthermore, prior work has demonstrated that training canines to find additional odors than necessary for their discipline, has no negative impacts on detection canine odor memory (9, 10).

These results highlight that training canines to a safe odor that can be placed in operational environments can have significant and important improvement on detection canine performance. Although prior research has utilized arbitrary odors such as vanillin, the same benefits would transfer to the UDC, which has the added benefit of being rare in operational environments. Thus, any instances of UDC in an operational environment are under the trainer control in contrast to what might be expected with vanillin (51) which could appear in a variety of foods, perfumes, and scented materials in a real-life environment.

### 5.7 Odor sensitivity and threshold

Another significant benefit of the UDC is that its concentration is readily manipulable, which can be challenging for traditional target odor materials; Since recent data suggests that canines must be trained on varying concentrations of a target odor in order to find large/bulk quantities and small/trace amounts of odor operationally (27–30), this feature is especially critical. Although training to a range of concentrations is important for all targets, the UDC and its relative ease of manipulation of concentration, may allow for training canines to engage in different search patterns based on odor volatility. For example, in prior biomedical detection canine research, canines were taught to respond to and discriminate between patient samples that were positive or negative for SARS-CoV-2. The UDC was utilized to train the canines to detect low quantities of odor in preparation for distinguishing between patient samples with assumed low odor concentrations. To accomplish this, we varied the concentration of the UDC from a relative 1000X down to 0.001X and every order of magnitude between (i.e., 100X, 10X, 1X, 0.1X, and 0.01X) (unpublished). Training in this manner allowed us to challenge the canine's olfactory threshold and ensure they were diligently sniffing in order to perform the demanding task of biomedical detection. This method of enhancing detection performance by the systematic use of decreasing concentrations of an odorant in training has been shown to lower canine thresholds (i.e., increase sensitivity) in testing (30).

### 5.8 Detector canine selection

The UDC can be used to assist handlers, trainers, and directors during the process of detector canine selection. Often, canine selection is conducted using a standardized set of tests to assess behavior, environmental soundness, drive, and motivation. The American National Standards Institute (ANSI) American Standards Board (ASB) Standard 085, First Edition (2021) “Standard for Detection Canine Selection, Kenneling, and Healthcare” is an example of such requirements for selection testing (52). Most frequently, scent detection abilities are not explicitly tested, or if they are, they are tested using the canine vendor's training aids or toys that are likely contaminated. It would be helpful to standardize an odor detection assessment to incorporate into selection tests so that a canine's detection abilities can be tested and even compared to other canines within the selection pool. Candidate canines could be eliminated if they do not perform up to the standard set by the canine selection team, thus saving time and money associated with training. It should be noted, however, that detection proficiency of a single odorant like 1-BO does not necessarily correlate with detection proficiency of more complex odor profiles and vice versa, some canines may demonstrate excellent detection proficiency of complex odor profiles and struggle with behavioral false responses when searching for the UDC.

Aside from testing and comparing detection abilities, the UDC could also be used to verify detection training whereby the vendors train their canines on the UDC, and the selection team can bring their own UDC training aids to ensure that the canines have been properly trained. Currently, if the selection team wants to test a canine's detection abilities, they are often restricted to using the vendor's training aids. This renders the selection team unable to accurately verify the credibility of the canines' responses to odor.

### 5.9 Performance assurance

In olfactory studies where human assessors are used to evaluate industrial environmental odor contamination/pollution, it is critical to have standardized protocols. These protocols often require that the human assessor complete testing with respect to a standard reference odor (n-butanol) to ensure the assessor's sensitivity and performance and provide a reference value for comparing to other odors (53, 54).

In a similar fashion, the UDC can be used to provide assurance that the canine is functioning as intended and able to perform olfactory tasks, before deployment, during operations to maintain operational effectiveness, after an illness or injury, while canines are taking certain medications, and in austere environments or weather conditions. As previously mentioned however, one must caveat this type of UDC usage with the fact that it is a single chemical odorant and while a canine could reasonably be able to detect it whilst partially incapacitated, the canine's detection of more complex odor profiles could be hindered. Analogous to how we are able to calibrate our handheld detectors and laboratory instruments, we need to be able to ensure that our detection dogs are “on”, “working”, and calibrated. Currently, there is no standard practice to confirm that our canines are functioning, so we are often relegated to blind faith.

One question remains, why not use one of the canine's training aids for this purpose? Because (1) many training aids are rarely cycled out of rotation despite frequent use and handling making them subject to contamination and (2) they are often solid materials and difficult to manipulate concentration in a standard way. In general, an issue with canine training aids is the large variation between training aids. Indeed, there is batch-to-batch and within-batch variability of training aid odor profiles from the same manufacturer (31). If one utilizes true material of drugs or explosives, the canine community is a secondary customer. In the explosives realm, the primary customer is the mining industry and the specifications they have for product is that it is relatively stable and will explode upon detonation. In the drug/narcotics realm, the primary customer is the end-user who is using the drugs for their mind-altering effects. Neither explosive nor drug detection primary customers are concerned with the odor profile of the products they are using. When odor is not part of the criteria for a product to pass inspection and not a priority, then that end-product is not as useful to the canine community. Explosive and drug training aids can be legally procured through a couple of legitimate means for canine training; however, these training aids are often riddled with the same issues—the odor presented to the canine is contaminated with impurities, cross-contaminated with human scent or other training aid odors. These factors make most canine training aids difficult to use as reference materials for standard procedures and assessment. The UDC alleviates many of these issues by being inexpensive, easily replaceable, easy to manipulate concentration, and one pure chemical (in comparison to the complex chemical mixtures of most targets) to make standardization readily achievable.

## 6 Role in research, development, test, and evaluation (RDT&E)

Due to the inherent qualities of a UDC outlined in Table 1, a UDC is an attractive choice for ensuring the repeatability of canine detection and behavior experiments.

Mendel et al. (2018) undertook a study to evaluate the potential for canines to detect Laurel Wilt Disease. Laurel wilt, caused by the fungus *Raffaelea lauricola*, decimated more than 300 million laurel trees in the United States. Detection canines were first trained to detect the UDC, then trained and tested with samples of laurel wilt-infected avocado wood or pure cultures of *R. lauricola*. The role of the UDC in this study, was not only as an initial training tool, but also a way to conduct the majority of the search-locate-report training chain using an innocuous substance like 1-BO instead of conducting the training with potentially hazardous/infectious samples of pathogen. Thus, the study demonstrated the utility of UDC training in RDT&E and confirmed the ability of canines to provide early detection of the laurel wilt-infected pathogen in time to inoculate or cull the trees to prevent the spread of disease (55).

Some of the first COVID-19 detection canines were initially imprinted on the UDC at research groups at University of Pennsylvania and Florida International University (FIU) (44, 56, 57). In a study, four canines were initially trained to detect UDC on a scent wheel over the course of a month, training several days a week. The UDC was then removed and replaced with COVID-19 patient masks sterilized by UV-C light, resulting in an overall accuracy of canine detection of COVID-19 of 97.5% in 40 double-blind trials (42). In a second COVID-19 study nine canines were initially trained on UDC to train the canines on the mechanics and behavior of search of a scent wheel and then urine and saliva samples inactivated by detergent or heat. The canines were able to successfully discriminate between infected and uninfected samples regardless of the inactivation protocol (30). Finally, in a third COVID-19 study, five canines were initially trained on wheel searching mechanics using the UDC and then were trained to discriminate COVID-19 expression in body odor from T-shirts with the two highest performing canines able to distinguish the COVID-19 positive and negative individuals with an average performance of 88% sensitivity and 95% specificity (43).

Another study demonstrated canines' ability to detect sinonasal-inverted papilloma (SNIP) after initial training with UDC. Four canines were trained to detect UDC searching an eight-port circular scent wheel and then were trained to detect SNIP within blood plasma; the canines were also able to generalize the SNIP odor and detected SNIP in nasal secretions without additional training (58). UDC has also been used to assess impacts on canine olfactory capabilities including demonstrating that canine ability to detect UDC was not affected by intravenous fentanyl sedation followed by naloxone reversal. In this study 10 canines were able to detect UDC at the lowest concentration of UDC available (0.27 ng/min) with extremely low false alerts (two false alerts across 80 trials) and then sedated with 0.3 mg of fentanyl citrate intravenously and after 10 min received 4 mg naloxone either intranasally or intramuscularly. All of the dogs were able to perform their searches for UDC and narcotic odors at levels comparable to pre-sedation two-hours, 24 h and 48 h post-sedation suggesting that IV sedation and subsequent intranasal or intramuscular naloxone does not affect dogs' abilities to search their target odors (59).

**Figure 5:**
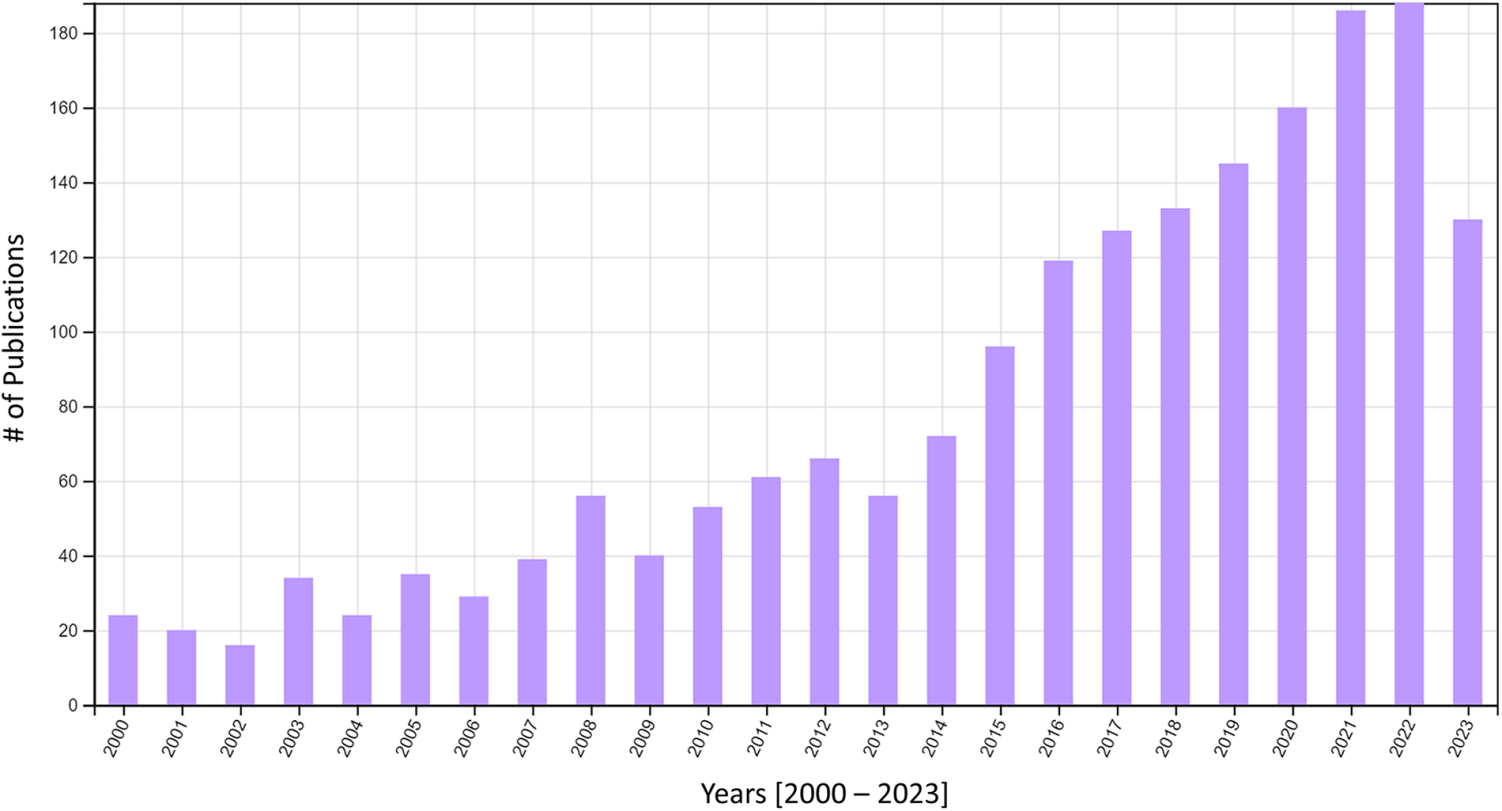
Number of publications by year in canine detection science. Data were derived from the Web of Science Core Collection Database using the following search terms: ((((ALL = (canine OR dog AND detection AND olfact* AND odor)) AND (**DT**==(“ARTICLE” OR “PROCEEDINGS PAPER” OR “REVIEW” OR “EARLY ACCESS” OR “BOOK CHAPTER”) AND **TMSO**==(“3.232 Veterinary Sciences” OR “7.262 Explosives” OR “3.220 Smell & Taste Science” OR “3.51 Dairy & Animal Sciences” OR “3.274 Animal Sensing”) AND **TMIC**==(“3.220.701 Olfaction” OR “3.220.1595 Electronic Nose” OR “7.262.2024 TNT” OR “3.232.1375 Animal-Assisted Therapy”) AND PY = (2000-2024))))). Search was completed on 2 January 2024. Results were refined by Document Type represented in the search term “DT”, Citation Topics Meso represented in the search term “TMSO” and Citation Topics Micro represented in the search term “TMIC”. Refinement was conducted to eliminate irrelevant publications on detection of canine medical conditions. Citation Report graphic is derived from Clarivate Web of Science, Copyright Clarivate 2024. All rights reserved. Full report of individual publications is available in Supplementary Data.

Widespread use of the UDC can help to standardize and unify much of the canine olfaction research being conducted worldwide. Canine detection RDT&E is still in its infancy with respect to research activity and peer-reviewed publication. We believe this is partly due to the fact that the largest detection canine programs in the United States are housed within U.S. government agencies (e.g., Transportation Security Administration (TSA), Customs and Border Protection (CBP), Federal Protective Service (FPS), United States Department of Agriculture (UDSA), Bureau of Alcohol, Tobacco, and Firearms (ATF), and the Military Working Dog enterprise spanning Army, Air Force, Navy, Marine Corps, and Coast Guard) and local, county, and state law enforcement agencies. These agencies have an operational mandate, but not a research mandate, therefore their priorities are focused on training and deploying canines for detection and/or patrol work. The rest of the detection canines in the U.S., represent a small fraction of the aforementioned detection canines and have a similar focus on operations over RDT&E. What remains are the small population of canines used in RDT&E conducted primarily by academic institutions. To this end, there have been only 1,909 publications (document types were peer-reviewed articles, proceedings papers, review articles, early access articles, and book chapters) on detection canine science within the past 23 years worldwide (Figure 5). The Figure below illustrates the growing field of canine detection science. The use of standard protocols to train detection dogs using the same odorant, i.e., the UDC, could help improve replicability across laboratories limiting variability due to the odor source alone. These efforts would go a long way to addressing the conclusions raised by Lazarowski et al. (2021), “lack of standardization in canine olfactory detection assessments, both in scientific research and in evaluations of operational canines, has led to a wide variability in results…attempts should be made to increase consistency in methodologies, such as standards for necessary controls to include and reporting of data, to allow for ease of interpreting results, internal validity of data, and making meaningful comparisons across studies” (60).

The UDC can be used in testing operational canines. Many canines that are ideal candidates for RDT&E will eventually become operational canines. Meaning, one must carefully consider the odorants to which the canine is trained, as this can impact the utility of these canines in future operations. This can significantly hamper experimental design if testing must be limited to odorants that a canine may have as potential future target odors. UDC helps release some of these constraints to allow for more flexible experimental designs, by being an unrelated odor that will have minimal impact on that canine's future employability as an operational detection canine.

Having an odor unrelated to future detection work gives Experimenters new possibilities to enhance experimental design. This includes pre-training canines prior to an experiment to assess baseline detection performance capabilities or even detection sensitivity limits. Such procedures are readily available in human olfactory work using n-butanol as a reference odor with olfactometer-based or Sniffin Stick threshold tests (2, 61, 62). After obtaining baseline detection sensitivity measures for each canine, the Experimenter can conduct block randomization (a procedure in which the assignment of treatments to experimental subjects and/or the order in which the experiment is done, are balanced by a control variable) to control for *a priori* differences in detection threshold or overall detection performance. Further, having an arbitrary odor or UDC for training allows Experimenters to ensure canines have equivalent detection training experience across experimental groups. This was recently leveraged in a research study by DeGreeff et al. (31). Here, two groups had access to either a live target material (triacetone triperoxide; TATP) or a non-detonable training aid. The group trained with live TATP only had limited access to TATP due to the availability of the explosive and its custodians (the FBI), while the experimental group had unlimited access to their non-detonable training aid during training. To balance the groups in terms of the amount of odor detection training time received, the UDC was used as a training aid such that both groups had unlimited access to odor detection training. This helped bridge the gap between potential discrepancies in training time that could affect the outcome of the experiment (31).

The UDC also lends itself to be used as a positive control in operational testing environments. Positive controls are used to evaluate the validity of a canine detection test by ensuring that canines are responding to target odor (e.g., UDC) in the testing scenario. Positive controls are employed extensively in the testing of biological sensors, laboratory instruments, and handheld detectors and are a critical component of scientifically sound experimental design. Having a standard reference odor for a positive control in experimental tests of canine performance would be a valuable additional research tool.

Future work to build a range of UDCs would also be valuable for research evaluating canine detection of a range of odors and/or odor memory. For example, one potential use of a range of UDC odorant concentrations would be to more precisely evaluate the effect of age on olfaction and associated cognitive decline (63). Tests, such as the University of Pennsylvania's Smell Identification Test (UPSIT) for humans could be adapted for canines utilizing a range of UDCs that allow for the longitudinal assessment of operational canines. Development of such a tool could go a long way in helping researchers standardize canine assessment which would allow for better comparison and meta-analysis of results across institutions, time, and canine age groups (64).

## 7 Conclusions

In this review, a universal detector calibrant and its applications in training and testing was discussed. Due to the numerous mandatory criteria that a candidate UDC odorant must meet, there is currently only one odorant in use as a UDC, 1-Bromooctane. Inclusion of the UDC in detection studies can help to standardize and unify canine olfaction research. UDC criteria listed herein has the potential to lead development of additional UDC odorants resulting in an array of calibrants and options to use in canine training and testing. Of all the senses, olfaction is the least studied and most poorly understood. A significant investment in olfactory sciences, to include instituting UDCs and other standardized protocols, will help close the gap in our understanding of the remarkable abilities of canine scent detection.
